# 

**Published:** 1911-04-08

**Authors:** 


### Tincture of Black Pepper.

The difficulty of finding an alternative to alcohol for those who have been so long in the habit of dram drinking as to have produced a condition of alcoholic gastritis is familiar, and the value of tincture of capsicum in this respect is well established. Capsicum was recommended by Sir Lauder Brunton long ago, particularly for the relief of that sinking feeling in the pit of the stomach that occurs in dipsomaniacs. Sir James Sawyer has recently drawn attention in the *Prescriber* (vol. v., No. 52, p. 15) to an alternative remedy made from black pepper. His suggestion is based upon the fact that in some of the poorest districts of London those who, for one reason or another can no longer afford to purchase spirits have been in the habit of using an infusion of black pepper as an alternative. Sir James proposes the title of Tincture Piperis Nigri Fortis for the medicinal preparation, which is made as follows:—

Take of black pepper, in fine powder, 10 ounces; rectified spirit a sufficiency. Pack the pepper tightly in a percolator and pour over it carefully half a pint of the spirit. At the expiration of two hours add more spirit, and let it percolate slowly until one pint of the tincture has been collected. Dose, 5 to 20 minims.

### MEDICAL APPOINTMENTS, ETC.

Dr. S. Tucker, M.B., B.S. Lond., M.R.C.S. Eng., L.R.C.P. Lond., D.P.H., has been appointed house-physician at the General Hospital, Tunbridge Wells.

Mr. Gerald G. Wray, M.B., Ch.B. Vict., has been appointed a resident medical officer at the Manchester Children's Hospital.

Mr. J. J. S. Rowe, Charing Cross Hospital, has been appointed house physician to the Evelina Hospital for Sick Children.

### Gifts and Bequests.

Prince Alexander of Teck has received the following subscriptions and donations to the Prince Francis of Teck Memorial Fund for the Middlesex Hospital: Sir John W. Kelk, £52 10s.; Messrs. Debenham and Freebody, £26 5s.; the Duchess of Northumberland, £25; the Duke of Roxburghe, £25.

Prince Alexander of Teck has received 1,000 guineas from members of the Royal Automobile Club, to endow a bed at the Middlesex Hospital in memory of Prince Francis of Teck, and 100 guineas from Lord and Lady Newlands and £50 each from Lord Rosebery and Mr. J. C. Hanbury.

Mr. H. Wildt, a Life Governor has given £57 10s. to the Leicester Infirmary.

Mr. Eric Barff Charlesworth, barrister, who left estate of the gross value of £31,314, has left his interest in his father's property as to £1,000 to Addenbrooke's Hospital, Cambridge, for the Children's Ward.

Among the latest contributions for King Edward's Hospital Fund for London are: Mr. Howard Morley, £105; Messrs. Robarts, Lubbock, and Co., £100; Messrs. Frank Bailey and Co., £52 10s.; Lord Farquhar, £50; staff at Frederick Gorringe, Limited, £31 2s. 7d., annual subscriptions.

The executors of the will of Louisa Sophia Lady Goldsmid find themselves in a position to make a further distribution of £3,000, representing part of her residuary estate, and have allotted to each of the following charities £500: Royal Maternity Charity (Samaritan Fund), Hospital for Women, Soho Square, and Queen Victoria Jubilee Institute for Nurses.

Mr. Joseph Henry Houldsworth, racehorse owner and a member of the Jockey Club since 1874, left, in addition to real estate of considerable value, £327,507. Among his bequests were a life annuity of £50 to Sister Mary, of the London Homœopathic Hospital, Great Ormond Street, for the valued attention and kindness bestowed by her during his several illnesses. He also left £5,000 to the London Homœopathic Hospital; £1.000 each to the Glasgow Royal Infirmary, the Western Infirmary, Glasgow, the Victoria Infirmary, Glasgow, the Glasgow Royal Hospital for Children, the Broomhill Home for Incurables, the Royal Scottish Hospital in London, and the Ayr County Hospital.

### Jottings.

### The British Hospitals Association.

The next Conference of the British Hospitals Association will be held in Manchester on the official invitation of all the hospitals of the city. The Lord Mayor has fixed Thursday and Friday, September 28 and 29, as the dates for the meetings.

Charles Edward Pearson, the superintendent fireman who was grievously and irremediably injured in the Sidney Street raid, is an accepted candidate for admission to the Royal Hospital for Incurables, Putney Heath. A donation of one guinea to the institution entitles the donor to four votes at the next Election, which will be held in May.

Mr. George Gray, the young Australian billiard-player, has promised to pay a visit to the Royal Hospital for Incurables, Putney Heath, and give an exhibition game there before the patients upon the table recently presented to the Institution by the Duchess of Northumberland.

The Queen has shown her sympathy with the work of the Metropolitan Asylums Board by giving her name to the Carshalton Hospital for Children, and consenting to its being styled the Queen Mary's Hospital for Children. This was announced by Mr. W. Dennis, chairman of the Managers of the Metropolitan Asylum District.

The London Homœopathic Hospital is appealing for £13,000 with which to build a home for the nurses.

Lord Duncannon will act as hon. secretary to the committee which is now being formed to arrange for a fancy-dress skating carnival in aid of the Prince Francis of Teck Memorial Fund at the Maida Vale Rink on May 1.

The plans for the new out-patient building at the Royal South Hants and Southampton Hospital, the cost of which was provided last autumn by Miss Burrell's generous gift of £7,000, are in a forward state and have been approved. During April probably tenders will be invited.

Mr. E. H. Phillips, hon. secretary of St. Mary's Hospital, Manchester, states that the appeal to women has been so successful that the hospital will be opened, debt free, probably in a fortnight. Mrs. Stewart Garnett is the hon. treasurer of the Women's Fund.

Prince and Princess Alexander of Teck have promised to attend a fancy dress ball and supper at the Savoy in aid of the Prince Francis of Teck Memorial Fund. The ball, which will be under the patronage of Princess Christian and the Duchess of Teck, will be held at the Savoy Hotel on Thursday, May 18, and the whole of the restaurant, the foyer, the winter garden, and the new ballroom are to be utilised for the occasion. At least 1,500 holders of tickets will be able to sit down to supper at the same hour. Fancy dress is indispensable. Domino cloaks will be allowed for gentlemen only, and may be hired at the hotel on arrival.

Prince and Princess Alexander of Teck will be present on Tuesday evening, April 11, at the opening of the Scala Theatre. The entire evening's receipts in full are to be given to the late Prince Francis of Teck's Fund for the Midlesex Hospital. The programme will introduce "Kinemacolor," an amazing process which reflects life in detail upon the screen in natural colours; and the opera by Herr Paul Lincke, of Berlin, entitled "Castles in the Air," adapted from "Frau Luna."

The preparation of the census papers at the Royal Hospital for Incurables, Putney Heath, reveals the average age of the 234 patients to be fifty-six.

A meeting, presided over by the Lord Mayor, was held at the Guildhall, Bristol, last week to aid the Committee of the Bristol General Hospital in a special appeal for £45,000 for a new wing to be known as the King Edward VII. Memorial Wing, and to consist of a female medical ward, a maternity ward, a new dental department, and additional administrative departments and offices. An endowment is also needed of £15,000. The Lord Mayor read a list of donations, including £10,000 given by the late Mrs. Procter-Baker, in memory of her husband, the late president. The list amounted to £33,735.

### The Hospital World.

### APPOINTMENTS VACANT.

Full particulars of these vacancies can be obtained on reference to our advertisement columns :

Bath Royal United Hospital.—House Surgeon.

Charing Cross Hospital.—Resident Medical Officer.

Ingham Infirmary, South Shields.—Senior House Surgeon, Junior House Surgeon.

Leicester Infirmary.—Assistant House Surgeon.

Liverpool Royal Southern Hospital.—Almoner and Out-Patients' Department Supervisor.

Prince of Wales' General Hospital, Tottenham.—Senior House Physician, Junior House Surgeon, Junior House Physician, Honorary Physician to *x*-Ray and Electrical Department.

Sheffield Royal Infirmary.—Junior Assistant House Surgeon.

Swansea General and Eye Hospital.—House Physician.

West Bromwich District Hospital.—Assistant Resident House Surgeon.

Princess Louise has given her patronage to two performances by the Garrick Amateur Dramatic Society of "The Prisoner of Zenda" on May 11 (evening) and May 13 (matinée) at the Royal Court Theatre, in aid of Lady Juliet Duff's appeal for Charing Cross Hospital.

### BUILDINGS CONTEMPLATED AND IN PROGRESS.

Carnarvon.—Mr. Rowland Lloyd Jones is to be the architect of the new hospital in connection with the workhouse here. Plans of two sites have been forwarded to the Local Government Board.

Colne (Lancs).—The Jubilee Cottage Hospital is to have twelve more beds through the generosity of Sir W. P. Hartley, if the endowment fund be increased by the amount of their cost.

Kirkcaldy.—Sir M. B. Nairn, Bart., has munificently offered to defray the cost of extending and equipping the Kirkcaldy Hospital. The extension alone is expected to cost £10,000.

London.—The lease of the Central London Ophthalmic Hospital, Gray's Inn Road, being about to expire, the committee has acquired a site in Judd Street, and building operations have been begun. A sum of £12,000, which includes a grant of £1,000 from King Edward's Hospital Fund, is in hand, but it is only sufficient to build to the third floor. A further £5,000 is required, and it is hoped that this sum will enable the whole building to be completed at one time.

Nottingham.—Mr. Arthur Marshall, A.R.I.B.A., is architect, and Messrs. Barlow and Sons are the builders of the new eye hospital which is being built at an estimated cost of £9,000 and will be completed in autumn. Accommodation will be provided for twenty-eight in-patients and there will be an out-patients' department.

Perth.—Mr. James Miller, A.R.S.A., F.R.I.B.A., of Glasgow, is architect for the new infirmary buildings at Taymount Terrace, for which tenders are being invited.

Seaton Moor.—The Parish Council of Seaton Moor has approved the plans of the Derwent Joint Smallpox Hospital Committee for the proposed new smallpox hospital.

Sheffield.—The extension work of the Sheffield Royal Infirmary is now completed, and will be occupied shortly. It comprises six large wards and twelve small isolation wards, and will accommodate nearly two hundred patients. When the patients can be removed to the new blocks certain alterations will be made to the old building to bring the whole establishment up to the modern standard. The total cost of the scheme is about £40,000, and the fund now stands at £33,500.

South Hants.—The plans for the new out-patient building at the Royal South Hants and Southampton Hospital, the cost of which was provided last autumn by Miss Burrell's generous gift of £7,000, are in a forward state and have been approved. In April probably tenders will be invited.

Stratford-on-Avon.—Mr. W. Henman, F.R.I.B.A., of Birmingham, is architect for the additions and alterations to Stratford-on-Avon Hospital. A tender of £2,450 by Messrs. Fincher and Co. has been accepted.

Worthing.—The new out-patient department at the Worthing Hospital has been designed by Mr. A. Morris Butler, F.R.I.B.A., and a contract has been accepted of £2,700. The work is to be completed by the end of September.

---

Sir William P. Hartley has offered to enlarge the Jubilee Cottage Hospital at Colne at a cost of £4,500. The hospital was erected by Sir William Hartley. There is attached to the gift a condition that the people of Colne shall subscribe an endowment fund of an equal amount to the cost of the enlargement.

